# Persistence of right ventricular dysfunction in infants after perinatal asphyxia treated with therapeutic hypothermia

**DOI:** 10.1038/s41390-025-03858-9

**Published:** 2025-01-15

**Authors:** Anna Foth, Willem-Pieter de Boode, Florian Kipfmueller

**Affiliations:** 1 Department of Pediatric Intensive Care, Our Lady’s Children’s Hospital, Dublin, Ireland; 2 Department of Neonatology, Radboud University Medical Center, Radboud Institute for Health Sciences, Amalia Children’s Hospital, Nijmegen, The Netherlands; 3 Department of Neonatology, University Children’s Hospital Mannheim, University of Heidelberg, Mannheim, Germany; 4 Department of Neonatology and Pediatric Intensive Care, Children’s Hospital, University of Bonn, Bonn, Germany

The pathophysiology of perinatal asphyxia is remarkably complex, triggering a cascade of biochemical dysregulations across multiple systems.^1,2^ The distinctiveness of neonatal asphyxia lies in its timing, taking place during the vulnerable window of postnatal adaptation where changes in blood oxygen levels play a pivotal role in pulmonary and cardiovascular transition.^3^

At the histological level, including the myocardium, hypoxia initiates VEGF overexpression, which activates angiogenic remodeling leading to endothelial apoptosis and disseminated intravascular coagulation.^4^ These alterations impair the endothelial barrier, which might lead to multiple organ injury, such as coronary artery congestion and thrombosis.^5^ Consequently, early after asphyxia, the cardiovascular system demonstrates high pulmonary vascular resistance (PVR), reduced systemic vascular resistance (SVR), and abnormal ductus arteriosus shunting, further impairing coronary perfusion and exacerbating myocardial ischemic injury.^6^

The right ventricle (RV) appears particularly vulnerable under these conditions. As the dominant, metabolically active ventricle during fetal life, its crescentic, thin-walled structure makes it more sensitive to afterload changes compared to the ellipsoid, thick-walled left ventricle (LV).^7^ At a high RV pressure, right coronary perfusion shifts from taking place in both systole and diastole to become diastolic-dependent. This may be further compromised by increased end-diastolic pressures.^8^ Moreover, a hypoxic-ischaemic cardiomyopathy leads to impaired myocardial contractility which further compromises the coronary perfusion. This situation can additionally deteriorate during therapeutic hypothermia (TH), which may induce bradycardia and increase PVR.^9^

Recent research has revealed intriguing aspects of cardiomyocyte remodeling in the neonatal period. Contrary to adult hearts, neonatal hearts demonstrate remarkable regenerative capacity in response to hypoxic-ischaemic injury. However, perinatal hypoxia may activate fetal programming through altered expression of cardioprotective genes, potentially increasing susceptibility to future ischemic injuries.^10^ Additionally, cardiac fibroblasts play a critical role in reprogramming RV myocytes, enabling the reactivation of a fetal-like phenotype over the long-term.^11^ While this initially supports adaptation to pressure overload, it may accelerate maladaptive responses over time. Enhanced cardiomyocyte proliferation has also been observed as a compensatory response to pressure overload.^12^ However, hypoxia-induced delays in sarcomeric assembly and isoform switching, combined with intracellular collagen content and activation of matrix metalloproteinases, can result in permanent impairment in cardiac performance.^13–15^

The report by Biran et al. in this issue of Pediatric Research provides insights on altered cardiac function at long-term follow-up in this population.^16^ The authors conducted a retrospective single-center study evaluating neonates with moderate to severe hypoxic-ischemic encephalopathy (HIE) who underwent TH between January 2014 and March 2023. The cohort was divided based on initial amplitude-integrated electroencephalography (aEEG) voltage patterns: normal (Group 1) versus abnormal amplitude (Group 2). Brain MRI was performed post-TH around day 10 of life, with descriptive results scored on a scale of 0–4 according to severity of hypoxic injury. All included neonates underwent echocardiographic assessment during their NICU stay and at a 6-month follow-up, incorporating comprehensive speckle-tracking echocardiography (STE) analysis for detailed evaluation of RV mechanics.

Initial NICU assessments revealed an RV dysfunction prevalence of 28% with 50% of cases demonstrating elevated PVR, with a comparable proportion between groups. Follow-up STE analysis at 6-months demonstrated that neonates with initially abnormal aEEG patterns exhibited enhanced RV contractile mechanics, characterized by more negative RV peak free wall longitudinal strain (−28.2 vs. −26.0%), RV peak global longitudinal strain rate (−1.24 vs. −1.10), and RV-peak free wall longitudinal strain rate (−1.50 vs. −1.27), while LV parameters and diastolic deformation remained comparable between groups.

The authors hypothesized that the observed hyperdynamic RV function may represent an adaptive response to increased afterload or early cardiac remodeling. These findings suggest that RV response to underlying processes could have long-term implications for cardiac health, even in the absence of LV dysfunction or markers of pulmonary vascular disease.^9^

The authors acknowledge methodological limitations, including inconsistent echocardiographic timing and the absence of standardized follow-up protocols for neonates with HIE. This may have introduced bias by disproportionately representing more critically ill neonates between groups and by limiting the retrospective echocardiographic interpretation.^12^ Furthermore, 53% of neonates received cardiovascular therapies, including adrenaline and dopamine, which could have influenced PVR and confounded these findings.

To address these limitations, the authors emphasize the importance of implementing standardized neonatal cardiovascular screening protocols, including neonatologist performed echocardiography, to optimize hemodynamic management and potentially improve long-term cardiovascular outcomes.^8^ Future studies should consider imaging modalities such as cardiac MRI or 3D echocardiography to assess subtle LV structural or functional changes, particularly given the possibility of preferential LV recovery.^17,18^

Another significant limitation of the study was the reliance on aEEG patterns and descriptive MRI interpretations without integrating comprehensive cerebrovascular monitoring. Future research should focus on multimodal neuromonitoring approaches, combining blood flow dynamics assessments, such as NIRS and resistance index (RI) via Doppler ultrasound, to better evaluate cardiocerebral interactions. Such methodologies would facilitate a deeper understanding of the complex heart-brain axis, from acute neonatal adaptation through to long-term pathways.

Emerging evidence highlights unique RV functional deviations in medium-term outcomes of HIE, contrasting with the traditional focus on LV dysfunction. Experience from populations of preterm infants, congenital diaphragmatic hernia, and congenital heart disease neonates suggests that RV dysfunction can fully recover in the absence of pulmonary vascular remodeling.^18^ However, in HIE, where pulmonary remodeling is frequently absent, RV dysfunction arises from a multifactorial pathophysiological cascade.

The observed differences in RV function at 6 months potentially represent either recovery dynamics or underlying pathology, underscoring the need for systematic echocardiographic assessment and neurodevelopmental monitoring in follow-up protocols.

Optimizing cardiovascular management requires increased attention to RV afterload modulation using vasodilator therapies, as RV recovery appears strongly linked to afterload reduction.^6^ Selecting inotropic support also demands consideration of the complex interactions between PVR, SVR, and myocardial oxygen demand. Individualized management strategies should be guided by neonatologist performed echocardiography and consider factors such as the severity of initial acidosis, specifics of resuscitation, and the presence and duration of prenatal hypoxia. This approach could identify different physiological phenotypes and refine management strategies.

The complexities of the heart-brain axis necessitate a cautious approach to aggressive inotropic support, with careful consideration of effects and side effects. The role of cardioprotective therapies in this setting remains unclear and warrants further investigation into their potential to mitigate maladaptive RV remodeling. Although the RV demonstrates remarkable regenerative potential, persistent dysfunction and remodeling may increase the risk of sudden death, impair adaptive ability, and increase intolerance to hypoxia.

In conclusion, while the findings of Biran et al. are promising, further prospective multicenter studies are warranted, implementing updated HIE classification guidelines with a focus on cardiocerebral interactions. Extended follow-up periods, particularly at 2 and 6 years of age, could elucidate our understanding of possible interactions between cardiovascular and neurodevelopmental outcomes.
